# Implicitly Priming the Social Brain: Failure to Find Neural Effects

**DOI:** 10.1371/journal.pone.0056596

**Published:** 2013-02-20

**Authors:** Katherine E. Powers, Todd F. Heatherton

**Affiliations:** Department of Psychological and Brain Sciences, Center for Cognitive Neuroscience, Dartmouth College, Hanover, New Hampshire, United States of America; University College London, United Kingdom

## Abstract

Humans have a fundamental need for social relationships. Rejection from social groups is especially detrimental, rendering the ability to detect threats to social relationships and respond in adaptive ways critical. Indeed, previous research has shown that experiencing social rejection alters the processing of subsequent social cues in a variety of socially affiliative and avoidant ways. Because social perception and cognition occurs spontaneously and automatically, detecting threats to social relationships may occur without conscious awareness or control. Here, we investigated the automaticity of social threat detection by examining how implicit primes affect neural responses to social stimuli. However, despite using a well-established implicit priming paradigm and large sample size, we failed to find any evidence that implicit primes induced changes at the neural level. That implicit primes influence behavior has been demonstrated repeatedly and across a variety of domains, and our goal is not to question these effects. Rather, we offer the present study as cautionary evidence that such a paradigm may not be amenable to scanning in an fMRI environment.

## Introduction

Humans have a fundamental need to belong to social groups [1], [2]. From an evolutionary perspective, the drive to form social connections may have evolved as an adaptive mechanism to promote survival, as group membership afforded the benefits of shared resources, security, and social support. Because rejection from social groups is especially detrimental, the ability to detect threats to social relationships and respond in adaptive ways is critical [3], [4].

Past research has shown that specific brain regions support detection of social threats. Studies examining neural responses during the experience of social rejection have consistently revealed activation in the anterior cingulate cortex (ACC), although some studies implicate more dorsal ACC regions [5], while others observed more ventral activations [6]. Other brain regions, including the ventrolateral prefrontal cortex and the insula, have also been shown to respond to social rejection [5], [7].

Equally as important as detecting social threats is the ability to respond in adaptive and profitable ways. Past research has demonstrated people may concurrently employ socially avoidant and affiliative strategies when social relationships are threatened [8], [9]. For instance, they attend to positive social information that might remedy their social distress and they simultaneously avoid negative social information that may exacerbate their distress [8]. Recently, we provided evidence of these simultaneous strategies by demonstrating that social rejection results in differential attempts to infer the mental states of others [10]. Specifically, the dorsomedial prefrontal cortex (dmPFC), a central component of the neural systems that support such mentalizing [11], [12], [13], was uniquely sensitive to positive social information, but was not engaged when viewing negative social scenes. Thus, the differential engagement of neural regions involved in understanding and empathizing with others depending on the valence of the surrounding social stimuli may subserve these behavioral responses.

Although there are reliable responses to explicit rejection [8], [14], much of our social perception and cognition occurs spontaneously and automatically [15], [16], [17]. Because social rejection is a major feature of human social life, dealing with potential threats of rejection may occur without conscious awareness or control. Therefore, constantly monitoring our environment for cues of social status and responding to these cues may be a relatively automatic process. Implicit priming has been widely used in behavioral research to activate particular concepts and stereotypes without participant awareness. For example, Sommer and Baumeister demonstrated that implicitly priming social rejection evoked differential behavioral responses on persistence and self-appraisal tasks, providing initial evidence that people respond to rejection cues automatically [18].

Interestingly, the specific pattern of avoiding negative and attending to positive social stimuli mirrors the well-documented “positivity effect” in older adults. That is, older adults tend to implement cognitive control strategies aimed at enhancing positive experiences and diminishing the effect of negative ones [19]. This intriguing parallel to the biased cognitions of excluded individuals (e.g., attending to positive stimuli and avoiding negative stimuli) led us to wonder if threatening belongingness needs might inadvertently prime elderly stereotypes by highlighting the loss of close, social connections, which older adults commonly experience as they grow older. To the extent that this is true, this general turning towards positivity should be reflected similarly for both rejected participants and those primed with an elderly stereotype.

Therefore, the present study had two interrelated hypotheses. First, we predicted that, following an implicit induction of social rejection, dmPFC would be preferentially engaged for positive social stimuli compared to negative, replicating our past work [10]. Second, we predicted that activating elderly stereotypes would produce a similar pattern of dmPFC activity. We tested these hypotheses in a between-subjects design with a large sample size, using an implicit priming paradigm that has been extensively used in behavioral research.

## Methods

### Participants

Eighty-four Dartmouth College undergraduates participated in this study. All participants were right-handed, had no history of neurological problems, and had normal or corrected-to-normal vision. They received course credit or were paid for their participation and gave informed consent in accordance with the guidelines set by the Committee for the Protection of Human Subjects at Dartmouth College. Eleven participants were dropped from the final analysis, due either to movement greater than 2.5 mm in any direction (n = 7) or poor fMRI signal quality (n = 4). This resulted in a total of 73 participants (37 female, age range 18–22 years).

### Implicit Priming Manipulation

The priming manipulation consisted of the scrambled sentence task that has been used previously to implicitly prime trait constructs [18], [20], [21], [22], [23]. Participants were instructed to form a grammatically correct short phrase or sentence consisting of three words from a string of four words, presented in a random order. Four versions of the sentence scramble test were compiled: rejection, acceptance, elderly, and neutral. Example trials from each condition include: *bullied spin school at* (rejection), *most voted popular for* (acceptance), *special to bird early* (elderly), and *lock strand gate the* (neutral). Each trial lasted for 8 seconds, and participants were instructed to press a button when they had successfully unscrambled the sentence. All conditions included a total of 40 trials. In the rejection, acceptance and elderly versions, there were a total of 20 critical priming stimuli intermixed with neutral items, a proportion (50%) that has been previously used to prime trait constructs, including social rejection [18], [21]. Participants were randomly assigned to a priming condition prior to their arrival. This resulted in a final count of 18, 18, 17 and 20 participants in the rejection, acceptance, elderly and neutral conditions, respectively.

### Procedure

Participants were informed that this study was examining the effect of personality on various cognitive processes. Once inside the scanner, all participants completed the priming manipulation described above. Immediately following priming manipulation, participants completed a 24-item mood questionnaire [10], [24] to assess subsequent changes in mood.

Participants then underwent functional magnetic resonance imaging while viewing a series of pictures selected from an online database. The pictures varied on dimensions of sociality (social, nonsocial) and valence (negative, neutral, positive), and were matched for arousal and extremity (e.g., distance of normative valence ratings from the midpoint of the rating scale) based on normative ratings obtained from a separate sample (N = 21) of participants. Example stimuli include: a funeral (social negative), buying groceries (social neutral), children playing at a water park (social positive), a burning building (nonsocial negative), a stack of books (nonsocial neutral), and a beautiful landscape (nonsocial positive). Critically, the nonsocial pictures did not contain any people. Each picture was presented for 2.5 seconds, and participants were asked to categorize each as an indoor or outdoor scene (a task chosen to ensure participants paid attention to all pictures, as well as to minimize the likelihood that they would infer the true purpose of the study). A total of 179 pictures were presented (30 per condition, with the exception of nonsocial positive, which only contained 29 pictures due to a programming error). The order of the pictures was pseudo-randomized and counterbalanced across participants. In order to accurately estimate the hemodynamic response function, pictures were intermixed with passive fixation trials of variable durations (0–7500 ms). To minimize interruptions following the priming manipulation, all pictures were presented in one functional run.

### fMRI Procedure and Analysis

Functional data were collected on a Phillips Intera Achieva 3 T scanner at Dartmouth College using an eight-channel phase arrayed coil (1 functional run consisting of 290 whole-brain volumes, 36 axial slices per volume, 3 mm thick, 0.5 mm gap, 3×3 mm in-plane resolution). An Epson ELP-7000 LCD projector was used to project stimuli onto a screen at the end of the magnet bore that participants viewed via an angled mirror mounted on the head coil.

Neuroimaging data were preprocessed and analyzed using SPM8 (Wellcome Department of Cognitive Neurology, London, UK). Preprocessing of functional data included slice time correction, realignment, unwarping, and normalization into standard space (3 mm isotropic voxels) based on the SPM8 EPI template that conforms to the ICBM 152 brain template (Montreal Neurological Institute, MNI). Normalized data were spatially smoothed using a 6 mm full-width-at-half-maximum Gaussian kernel.

A general linear model (GLM) incorporating six task regressors and covariates of non-interest (linear trend, six movement parameters derived from realignment) was specified for each participant. This GLM was convolved with a canonical hemodynamic response function (HRF) and used to generate contrast images comparing social to nonsocial activations for each participant. These contrast images were collapsed across all prime manipulation conditions and entered into a second-level random effects analysis, thresholded at *p*<.0001 with an extent threshold of 20 contiguously activated voxels. This analysis resulted in a whole-brain statistical parametric map identifying regions displaying greater activity to social than nonsocial scenes.

We performed a region-of-interest (ROI) analysis by centering a 6 mm sphere on the voxels of peak activation of regions identified by this contrast and extracting parameter estimates (β) for each participant. ROIs were thus defined in an unbiased manner, as all prime conditions contributed equally to the statistical parametric map used for ROI identification. Parameter estimates were submitted to offline statistical analyses in SPSS. Our primary analyses targeted our apriori defined brain region of interest, dmPFC. Supplemental analyses further examined how the primes affected regional brain responses to social scenes at the whole brain level.

## Results

### Behavioral Results

Analysis of the 24-item mood questionnaire indicated that the implicit prime had no effect on mood, *F*(3,69) = .19, *p* = .904; *M_rejection_* = 70.29, *SD* = 8.74; *M_acceptance_* = 68.21, *SD* = 8.08; *M_elderly_* = 68.90, *SD* = 12.60; *M_neutral_* = 70.34, *SD* = 1.20. This is consistent with prior behavioral research demonstrating that implicit primes do not produce changes in self-reported mood [25], [26].

### fMRI Results

**Figure 1:**
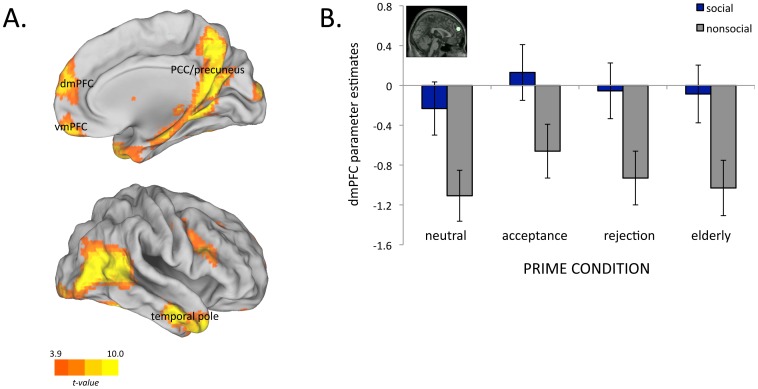
Neural responses following implicit primes. A. Results from a whole-brain, random-effects analysis of all participants contrasting social scenes to nonsocial scenes (*p*<0.0001, *k* >20), overlaid onto inflated cortical renderings. Results reveal the social brain, a network of regions including the medial prefrontal cortex, posterior cingulate cortex/precuneus and regions of the inferotemporal cortex that consistently respond to social stimuli. B. BOLD response of dmPFC to social compared to nonsocial scenes, showing no differences in activation across implicit prime conditions. Inset displays location of dmPFC ROI (MNI coordinates 3, 60, 27). Bars indicate standard error of the mean.

A whole-brain analysis comparing regions that displayed a greater response for social scenes compared to nonsocial scenes for all participants revealed a system of regions including the medial prefrontal cortex, posterior cingulate cortex/precuneus, and temporal poles (see Figure 1A). Prior research has demonstrated that these areas (e.g., the social brain) consistently respond to social stimuli [10], [17].

To investigate the effect of the implicit prime on the neural response to social scenes, parameter estimates from each ROI identified in this contrast were entered into a mixed-model ANOVA with prime condition (rejection, acceptance, elderly, neutral) as a between-subjects factor, and sociality (social, nonsocial) as a within-subjects factor. Analysis of our apriori defined brain region of interest, dmPFC (MNI coordinates 3, 60, 27), revealed no main effect of prime (*p* = .96) and no prime by sociality interaction (*p* = .72) (see Figure 1B). No other regions identified in the social>nonsocial contrast displayed a significant prime by sociality interaction (all *p*s >.53). That is, none of the implicit primes affected neural responses to social scenes.

Because we averaged neural activity across the voxels surrounding the peaks of the ROIs in the above analysis, we performed a supplemental between-subjects whole-brain ANOVA comparing across prime conditions, to ensure that subtle priming effects were not missed. Results revealed no significant clusters anywhere in the brain that survived correction for multiple comparisons (FWE, *p*<.05). Even at a more liberal threshold (*p*<.005 uncorrected, *k*>20), no significant clusters emerged.

Despite the robust finding that the implicit primes failed to modulate neural responses to social scenes, we further probed neural activity in dmPFC for effects of scene valence, to directly test our hypotheses. To do so, we computed a difference score for neural activity during social scenes relative to nonsocial scenes for each participant for all valence categories (negative, neutral, positive) [10]. A mixed-model ANOVA with prime condition as a between-subjects factor and valence as a within-subjects factor revealed no main effect of prime (*p = *.96) and no prime by valence interaction (*p* = .54). That is, dmPFC response did not vary as a function of scene valence across prime conditions.

## Discussion

The current study sought to investigate the automaticity of social threat detection and the parallel nature of preferential attunement to positive stimuli among those primed by feelings of rejection or by aging stereotypes by examining how implicit primes affect regional brain responses to social stimuli. Based on previous research, our primary analyses targeted dmPFC, as we hypothesized that particular patterns of activity in this brain region would be elicited by the implicit primes. Instead, dmPFC activity was not influenced by any of the primes. Supplemental analyses examining activity at the whole brain level confirmed that the implicit primes did not affect neural responses to social scenes.

Our predictions that priming effects would be observable in dmPFC are grounded in prior work investigating responses to explicit occurrences of social rejection. Behavioral research has demonstrated that social rejection motivates withdrawal from the surrounding social world [27], [28], [29] and antisocial behaviors [30], [31], and also that rejected individuals appear highly attuned to social information [32], [33], specifically that which is positive [8], [34], and display a propensity to engage in prosocial behaviors [34]. We previously demonstrated that these behavioral tendencies are reflected in dmPFC activity to social scenes following explicit interpersonal distress [10], and thus expected to observe dmPFC activity mirroring those responses when we used implicit rejection cues in the present study. Moreover, we expected priming elderly stereotypes would produce a similar pattern of dmPFC activity indicative of this general tuning towards positivity. Instead, we failed to find evidence that dmPFC activity was modulated by any of these primes. More broadly, we failed to identify any other brain regions showing enhanced activity in response to the primes.

Thus, despite using a well-established implicit priming paradigm and a large sample size, we failed to find any evidence that any of these primes induced changes at the neural level. Although prior work has demonstrated that implicitly priming rejection [18] and elderly stereotypes [21] elicit behavioral responses, our results suggest that these effects may be too subtle to be observed using fMRI. Again, our goal here is not to question the behavioral effect of implicit primes. We note that studies using this paradigm, as well as others, have repeatedly demonstrated behavioral outcomes across a range of domains, including social behavior [18], [21], [23], [25], [35], [36]. However, our results suggest that these effects may be too small to detect using fMRI, which typically requires large effect sizes, particularly in between-group designs. We acknowledge the lack of a behavioral dependent measure similar to those used in previous implicit priming studies here, and it remains a possibility (though one difficult to test) that the priming simply did not work. However, we note the consistency of our experimental protocol with prior work eliciting robust behavioral effects, as well as our replication of prior self-reported mood results following priming manipulations. Thus, although implicitly priming has provided invaluable contributions to our understanding of unconscious processes and behaviors, we offer the present study as cautionary evidence that such a paradigm may not be amenable to scanning in an fMRI environment.
